# Navigating complexity – how innovation shapes the future of surgery

**DOI:** 10.1515/iss-2025-0054

**Published:** 2025-12-02

**Authors:** Sabine Drossard, Juliane Kröplin

**Affiliations:** Klinik und Poliklinik für Allgemein-, Viszeral-, Transplantations-, Gefäß- und Kinderchirurge, Universitätsklinikum Würzburg, Würzburg, Germany; Oral and Maxillofacial Surgery, University Hospital of Rostock, 18057 Rostock, Germany

Surgery has always been defined by its capacity to adapt. Every generation of surgeons faces new dimensions of complexity and must develop innovative ways to master them. Each year, new technologies, refined techniques, and evolving concepts reshape how we operate, teach, and think about patient care. Yet true innovation does not arise from technology alone – it is driven by curiosity, collaboration, and the next generation of surgeons who challenge existing boundaries and reimagine what is possible.

With this third edition of *What’s New in Surgery*, Innovative Surgical Sciences once again brings together early-career surgeons from different specialties to highlight recent developments in their fields. The 2025 issue showcases four perspectives that span the breadth of modern surgical practice, from the educational challenge of mastering complex procedures in our smallest patients to the increasing demands of treating an aging population.

In their article “Recent advances in operative techniques for pancreatic surgery,” J. Strotmann and V. Tripke present three promising approaches to the management of pancreatic surgery. To address the complexity of these demanding operations, they discuss in particular the triangle operation, the use of autologous patches, and minimally invasive surgical approaches [1].

K. Klepka et al. discuss recent developments in the diagnosis, management, and outcomes of pelvic insufficiency fractures in their article “Pelvic insufficiency fractures in the elderly – what insights has the past year of research brought?”. Their review highlights the value of an interdisciplinary approach that combines precise diagnostics, individualized treatment strategies, and proactive osteoporosis management to optimize patient outcomes [2].

Implantology plays a special role in oral and maxillofacial surgery, requiring highly coordinated collaboration between dentists and surgeons. C. Friedrich et al. present promising digital approaches to planning and performing high-quality implantology in their article: “Current status and future developments in dental implantology – a narrative review” [3].

In pediatric surgery, S. Drossard and L. Schuffert reflect on how simulation-based training can support the acquisition of advanced operative skills in their contribution “The Role of Simulation in Mastering Highly Specialized Pediatric Surgery: Current Trends and Future Perspectives.” As surgical training transitions toward competency-based models, simulation has become an essential tool to ensure both proficiency and patient safety, bridging the gap between theoretical learning and clinical performance [4].

All articles share a unifying theme of complexity and transformation: each contribution addresses a field that has historically posed particular challenges, whether pancreatic resections, fragility fractures, dental implant reconstruction, or reconstructive surgery for congenital malformations. Together, these contributions reflect the dynamic landscape of surgical innovation – one that extends beyond technical skills to encompass education, patient safety, and interdisciplinary collaboration. The spirit of curiosity and cooperation embodied in this issue characterizes a new generation of surgeons who view innovation as a continuous process of learning and adaptation. As the boundaries of surgical practice continue to expand, we hope that *What’s New in surgery 2025* not only informs but also inspires readers to rethink the surgeon’s role in a rapidly evolving healthcare landscape.
